# Long intergenic non-protein coding RNA 847 promotes laryngeal squamous cell carcinoma progression through the microRNA-181a-5p/zinc finger E-box binding homeobox 2 axis

**DOI:** 10.1080/21655979.2022.2062531

**Published:** 2022-04-17

**Authors:** Wei Li, Xionghui Hu, Xiaolin Huang

**Affiliations:** a Department of Otolaryngology, Ezhou Central Hospital, Hubei, Ezhou Province, P.R. China; b Department of Otolaryngology, Tongxiang First People’s Hospital, Jiaxing, Zhejiang Province, P.R. China

**Keywords:** LINC00847, miR-181a-5p, ZEB2, LSCC, migration and invasion, proliferation

## Abstract

The present study is targeted at investigating the effects of long intergenic non-protein coding RNA 847 (LINC00847) on the malignant biological behaviors of laryngeal squamous cell carcinoma (LSCC) cells, and the mechanisms. Quantitative real-time PCR and Western blotting were conducted for detecting the expressions of LINC00847, microRNA-181a-5p (miR-181a-5p) and zinc finger E-box binding homeobox 2 (ZEB2) in LSCC cell lines and tissue samples. BrdU, cell counting kit-8, scratch wound healing, Transwell and flow cytometry assays were utilized for detecting cell proliferation, migration, invasion, and cell cycle progression. Dual-luciferase reporter gene, RNA binding protein immunoprecipitation (RIP), and RNA pull-down assays were utilized to investigate the interaction among LINC00847, miR-181a-5p, and ZEB2. The subcellular location of LINC00847 was determined by RNA fluorescence in situ hybridization (RNA-FISH) assay. Tumor growth was evaluated using a xenograft model of nude mice. It was revealed that LINC00847 expression was increased in LSCC tissues, and its high expression was associated with lymph node metastasis and poor differentiation. LINC00847 was mainly located in the cytoplasm of LSCC cells, and LINC00847 overexpression promoted LSCC cell proliferation, migration, invasion, and accelerated the cell cycle progression while knocking down LINC00847 had the opposite effects *in vitro* and inhibited the tumor growth *in vivo*. LINC00847 directly targeted miR-181a-5p and negatively modulated miR-181a-5p expression. ZEB2 was a target gene of miR-181a-5p, and was positively and indirectly modulated by LINC00847. Our data suggest that LINC00847 promotes LSCC progression by regulating the miR-181a-5p/ZEB2 axis.

## Graphical Abstract

**Figure:**
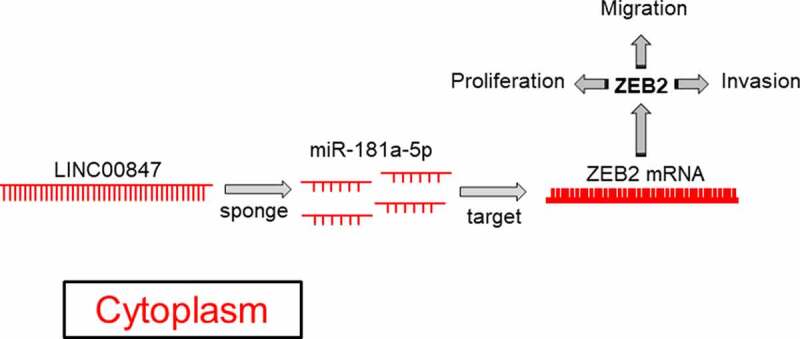

## Highlight

1. LINC00847 is highly expressed in LSCC tissues and cell lines;
2. LINC00847 expression level correlates with the prognosis of LSCC patients;
3. LINC00847 promotes the proliferation, cell cycle progression, migration and invasion of LSCC cells;
4. LINC00847 decoys miR-181a-5p and up-regulates ZEB2 in LSCC cells.

## 1. Introduction

Known as a head and neck malignancy, laryngeal squamous cell carcinoma (LSCC) has high morbidity, high mortality and low cure rate [1]. Clinically, according to pathological classification, LSCC cases are divided into glottic type, subglottic type and supraglottic type [2]. At present, the pathogenesis of LSCC remains unclarified, and it is believed to be correlated with human papillomavirus (HPV) infection, bad living habits, radioactivity, environmental factors and so on [3]. Surgery combined with chemotherapy/radiotherapy is the preferred therapy for LSCC patients, and although these treatment approaches can prolong the life of some patients and decelerate disease development, most patients have a very poor prognosis [4]. In this context, it is of great importance to clarify the pathogenesis of LSCC and look for novel treatment targets [5].

Long non-coding RNAs (lncRNAs) are characterized by over 200 *nt* in length, and they cannot encode proteins [6]. Previous research claims that lncRNAs are ‘noise’ generated during genome transcription [7]. However, in the last decades, it has been found that they can regulate multiple biological processes including tumorigenesis [8–12]. For instance, SNHG1 expression is elevated in cervical cancer (CC) cells and tissues, and down-regulation of SNHG1 *in vitro* can significantly restrain CC cell proliferation, migration and invasion [9]. It has been reported that long intergenic non-protein coding RNA 847 (LINC00847) is implicated in microcystin-LR-induced hepatotoxicity and the tumorigenesis of renal cancer [12,13]. Nevertheless, its biological functions and mechanisms in LSCC are not yet deciphered.

MicroRNA-181a-5p (miR-181a-5p) can suppress the progression of LSCC [14], while zinc finger E-box binding homeobox 2 (ZEB2) can facilitate the progression of LSCC [15]. In the present work, bioinformatics analysis showed that LINC00847 could probably directly target miR-181a-5p, and miR-181a-5p could probably target ZEB2 3ʹUTR. We hypothesized that LINC00847 had the potential to be a diagnostic biomarker and therapeutic target for LSCC. In this study, we investigated the biological functions and mechanisms of LINC00847 in LSCC progression, and demonstrated that LINC00847 promoted LSCC progression through targeting miR-181a-5p/ZEB2 axis.

## 2. Materials and methods

### 2.1 Tissue sample collection

Ethical approval (No. WJ2017LL26) was obtained from the Ethics Committee of Ezhou Central Hospital and the informed consent was obtained from participants. Fifty-five pairs of LSCC and para-cancer tissues were collected after the operation. All cancer tissues were pathologically confirmed as LSCC, and no other malignancies were found. None of the patients received chemotherapy or radiotherapy before the surgery.

### 2.2 Cell culture *[16]*

Human bronchial epithelial cells (16HBE) and LSCC cell lines (HEp-2, AMC-HN-8, TU177, and TU212) were bought from American Tissue Culture Collection (ATCC) (Manassas, VA, USA). These cells were cultured in Dulbecco’s modified Eagle’s medium (DMEM; Invitrogen, Carlsbad, CA, USA) containing 100 U/ml penicillin (Invitrogen, Carlsbad, CA, USA) and 0.1 mg/ml streptomycin (Invitrogen, Carlsbad, CA, USA) and 10% fetal bovine serum (FBS; Invitrogen, Carlsbad, CA, USA) at 37°C in 5% CO_2_. When the cells reached the logarithmic growth phase, they were collected for subsequent experiments.

### 2.3 Cell transfection *[17]*

HEp-2 and TU212 cells were inoculated into 60-mm culture plates (1 × 10^6^ cells/ml), which were then cultured in 5% CO_2_ at 37°C. After 24 h, cell transfection was conducted. The empty vector (NC), LINC00847 overexpression plasmid (LINC00847), ZEB2 overexpression plasmid (ZEB2), small interfering RNAs targeting LINC00847 (si-LINC00847-1 and si-LINC00847-2) and the negative control (si-NC), miR-181a-5p inhibitors, miR-181a-5p mimics, and their controls inhibitors NC and mimics NC were bought from Guangzhou RiboBio Co., Ltd. (Guangzhou, China). Following the manufacturer’s instruction for Lipofectamine® 2000 kit (Invitrogen, Carlsbad, CA, USA), the transfection was performed. Quantitative real-time PCR (qRT-PCR) was carried out 24 h after transfection to measure the transfection efficiency.

### 2.4 qRT-PCR *[18]*

TRIzol reagent (Invitrogen, Shanghai, China) was employed for total RNA extraction from cultured cells or LSCC tissues. A Reverse Transcription Kit (Takara, Dalian, China) was employed for the reverse transcription of RNA into cDNA. qRT-PCR was performed using a SYBR Green PCR kit (Takara, Dalian, China) on an Applied Biosystems 7500 Real-Time PCR System (Applied Biosystems, San Francisco, CA, US). The relative expressions of LINC00847, miR-181a-5p and ZEB2 mRNA were calculated by the 2^−ΔΔCt^ method, with β-actin and U6 as the internal references. The primers for qRT-PCR analyses are as follows (F for forward; R for reverse). LINC00847 primer sequence: F, 5’-AACGCTGCCTCTGTGGAAGTCTC-3’; R, 5’-CGCTCTGCTCTCCCGCCATC-3’. miR-181a-5p primer sequence: F, 5’-CTCGCTTCGGCAGCACA-3’; R, 5’-AACGCTTCACGAATTTGCGT-3’. ZEB2 primer sequence: F, 5’-GCAGTGAGCATCGAAGAGTACC-3’; R, 5’-GGCAAAAGCATCTGGAGTTCCAG-3’. U6 primer sequence: F, 5’-AAAGCAAATCATCGGACGACC-3’; R, 5’-GTACAACACATTGTTTCCTCGGA-3’. β-actin primer sequence: F, 5’-CACCTTCTACAATGAGCTGCGTGTG-3’; R, 5’-TAGCACAGCCTGGATAGCAACGTAC-3’.

### 2.5 RNA fluorescence in situ hybridization (RNA-FISH) assay *[19]*

RNA-FISH assay was performed to determine the subcellular location of LINC00847. In brief, TU212 cells were fixed in 4% formaldehyde for 15 min at room temperature, washed with phosphate buffer saline (PBS), and then permeabilized with 0.5% Triton X-100 on ice for 10 min. Next, the cells were washed with PBS, rinsed in 2× saline sodium citrate (SSC), and dehydrated with gradient concentration ethanol (70, 85, and 100%). Subsequently, biotin-labeled NC (Bio-NC-probe), LINC00847 (Bio-LINC00847-probe), or LINC00847-MUT (Bio-LINC00847-MUT-probe) were mixed with 40 μL of streptavidin agarose beads and incubated on a rotator overnight. Subsequently, the cells were incubated with the probes. After washing with hybridization buffer and 2× SCC, the cells were incubated with DAPI staining solution at room temperature in the dark. Finally, the LSM 800 confocal microscope (Carl Zeiss, Germany) was utilized to capture the images.

### 2.6 Cell counting kit-8 (CCK-8) assay *[20]*

The cells during exponential growth in each group were collected to make single-cell suspension. The cell density was adjusted after cell counting, and the cells were inoculated in the 96-well plate (2000 cells/well). The next day, each well was added with 90 μL of medium and 10 μL of CCK-8 solution (Dojindo Molecular Technologies, Dojindo, Japan). After incubating for 2 h, a microplate reader was adopted for determining the absorbance value at 450 nm of each well, and the data were recorded. For 4 consecutive days, the absorbance value of the cells were measured every 24 h. The cell growth curve was plotted with time as the x-coordinate and optical density (OD) at 450 nm as the y-coordinate.

### 2.7 BrdU assay *[21]*

HEp-2 and TU212 cells were transferred at 2.5 × 10^5^ cells/well to 24-well plates (with a cover slide in it). BrdU solution (Beyotime Biotechnology, Shanghai, China) was added after 24 h of culture, and the culture was continued for 4 h. Then, the cells were washed with PBS, and fixed for 30 min with 4% cold paraformaldehyde; the supernatant was discarded and 1× Apollo (100 μL) was added to each well for cell staining for 30 min; the supernatant was discarded and each well was added with 1× Hoechst 33,342 reaction solution (100 μL), and the cells were incubated for 20 min in the dark at room temperature. After PBS washing, in 10 randomly selected fields, the number of BrdU-positive cells and the total number of cells were counted under the microscope, and the percentage of BrdU-positive cells was calculated.

### 2.8 Scratch wound healing assay *[22]*

An appropriate number of transfected HEp-2 and TU212 cells were inoculated into 6-well plates, and complete medium (2 ml) was added to each well. When the confluence reached 80–90%, a pipette tip was employed for scratching the cells perpendicularly. After washing twice with PBS, the scratches were observed and photos were taken under an inverted microscope, and this time point was defined as 0 h. The cells were then cultured at 37°C in 5% CO_2_ for another 24 h. The healing of scratch wounds was observed at the same observation point. Scratch healing rate was calculated with (0 h scratch width – 24 h scratch width)/0 h scratch width as the migration capability of cells.

### 2.9 Transwell assay *[23]*

Fifty (50) μL of Matrigel was diluted and spread on the filter of the Transwell chamber. After air drying at 4°C, HEp-2 and TU212 cells were trypsinized and then centrifuged for 3 min at 1000 r/min. Serum-free DMEM was employed to resuspend the cells to adjust the cell density to 1 × 10^5^ cells/ml. Then, the upper compartment was loaded with cell suspension (200 μL), and 700 μL of medium with 10% FBS was added to the lower compartment. The cells were then cultured for 24 h in 5% CO_2_ at 37°C. Next, the chamber was taken out, and cotton swabs were utilized to gently wipe off the cells on the surface of the upper chamber. These cells were air-dried, stained for 30 min with crystal violet solution, and cleaned twice with PBS. Ultimately, the cells were observed and photographed using a microscope. The cells were counted in 5 randomly selected fields (×100), and for the number of invading cells, the average value was calculated.

### 2.10 Flow cytometry *[24]*

Cell cycle was analyzed by conducting flow cytometry. LSCC cells of two groups during logarithmic growth were inoculated at 1 × 10^4^ cells/well into 96-well plates. These cells were rinsed twice with PBS after 24 h of culture, fixed with 70% ethanol, and stored overnight at 4°C. The cell density was adjusted to 1 × 10^6^ cells/ml after rinsing once with PBS. Propidium iodide staining solution was added to make a final concentration of 0.05 mg/ml. Subsequently, the cell staining was performed for 30 min at 4°C. The flow cytometer was utilized to analyze the cell cycle distribution in the two groups. To exclude the cell debris, FSC v.s. SSC figure was used (R1); To exclude the dead cells, FSC v.s. PI figure was used (R2). Then the cell distribution in different phases was showed, and abscissa showed the DNA content, and ordinate showed the number of LSCC cells.

### 2.11 Xenograft assay *[25]*

Nude mice (4–6 weeks old, male) were obtained from the Experimental Animal Center of Wuhan University (Wuhan, China). All procedures of animal experiments were approved by the Ethics Committee of Ezhou Central Hospital. The above mice were kept under standard indoor conditions (23°C, 40% humidity, 12 h/12 h light-dark cycles, and food and water available ad libitum). The mice were randomly divided into four groups (control group, si-NC group, si-LINC00847-1 group, and si-LINC00847-1+ ZEB2 overexpression group) and injected subcutaneously with 1 × 10^7^ TU212 cells. After the injection, the subcutaneous tumors were measured using a vernier caliper every 7 days. The tumor volume was calculated as follows: V = (L × W^2^)/2 (V, volume; L, length; W, width). After 3 weeks, the mice were euthanized with 100% oxygen/5% isoflurane and sacrificed.

### 2.12 Dual-luciferase reporter system *[26]*

TargetScan (http://www.targetscan.org/vert_72/) and StarBase (http://starbase.sysu.edu.cn/) were employed for predicting the binding sites between miR-181a-5p and LINC00847, as well as between miR-181a-5p and ZEB2 mRNA 3ʹUTR. LINC00847 or ZEB2 3ʹUTR fragments containing the binding sites were amplified and inserted into the pmirGLO reporter vectors (Promega Corp., Madison, WI, USA) to construct wild-type LINC00847 (WT LINC00847), wild-type ZEB2 (WT ZEB2), mutant LINC00847 (MUT LINC00847) and mutant ZEB2 (MUT ZEB2) dual-luciferase reporter vectors. The above-mentioned reporter vectors, together with miR-181a-5p mimics or mimics NC and miR-181a-5p inhibitors or inhibitors NC, were co-transfected into HEp-2 and TU212 cells, respectively. The luciferase activity was determined based on the manufacturer’s instructions 48 h following transfection. The result was the ratio of the luminescence intensity of Renilla luciferase to that of firefly luciferase to reflect the binding intensity between miR-181a-5 and LINC00847, as well as between miR-181a-5p and ZEB2.

### 2.13 RNA immunoprecipitation (RIP) assay *[27]*

Based on the manufacturer’s instructions, RIP assay was conducted employing a Magna RIP RNA-Binding Protein Immunoprecipitation Kit (Millipore, Billerica, MA, USA) to verify the interaction between LINC00847 and miR-181a-5p. TU212 and HEp-2 cells were lysed with RIP lysis buffer, and cell lysate (100 μL) and magnetic beads conjugated with human anti-Argonaute2 (Ago2) antibody or negative control IgG were incubated in RIP lysis buffer. The immunoprecipitate was then incubated with proteinase K to remove the proteins. Subsequently, RNA was extracted. Next, qRT-PCR was conducted to detect miR-181a-5p and LINC00847.

### 2.14 Western blot analysis *[28]*

RIPA lysis buffer (Beyotime Biotechnology, Shanghai, China) containing protease inhibitors was utilized to lyse the LSCC cells, and the supernatant was collected after high-speed centrifugation, and the supernatant was heated for 10 min in a water bath at 100°C for protein denaturation. After protein quantification utilizing a BCA protein assay kit (Beyotime Biotechnology, Shanghai, China), sodium dodecyl sulfate-polyacrylamide gel electrophoresis (SDS-PAGE) was conducted and the proteins were transferred to polyvinylidene fluoride (PVDF) membranes. Next, after membrane washing with tris buffered saline tween (TBST), the membrane was incubated overnight with rabbit anti-β-actin antibody (ab8227, 1:1000) and rabbit anti-ZEB2 antibody (ab138222, 1:1000) at 4°C. After the rinse with TBST, the membranes were incubated with the secondary antibody goat anti-rabbit IgG H&L (HRP) (ab205718, 1:500) at room temperature for 1 h and then rinsed again with TBST, and the protein bands were visualized using an ECL chemiluminescence detection kit (Amersham Pharmacia Biotech, Little Chalfont, UK).

### 2.15 Statistical analysis *[29]*

SPSS 22.0 statistical software (SPSS Inc., Chicago, IL, USA) was applied to analyze the experimental data. Measurement data were expressed as ‘mean ± standard deviation’. Student’s t-test was used for the comparison between two groups, and one-way ANOVA was used for comparison among multiple groups. The count data were represented by a contingency table, and χ^2^ test was conducted for analyzing the difference between constituent ratios. A difference was statistically significant when *P* < 0.05.

## 3. Results

We hypothesized that LINC00847 could promote the progression of LSCC. Gain-of-function and loss-of-function models were established, and it was revealed that LINC00847 could regulate the malignant biological behaviors of LSCC cells. Additionally, it was demonstrated that LINC00847 modulated miR-181a-5p/ZEB2 axis, and the overexpression of miR-181a-5p reversed the effects of LINC00847 on the proliferation, migration, invasion, and cell cycle progression of LSCC cells.

### 3.1 The expression characteristics of LINC00847 in LSCC tissues

**Figure 1.**
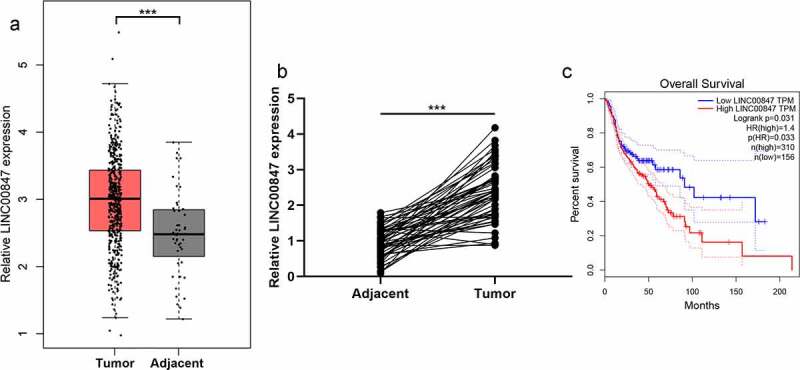
**LINC00847 expression is up-regulated in LSCC tissues** A. LINC00847 expression characteristics in LSCC tissues and adjacent tissues were analyzed using the GEPIA database. B. qRT-PCR was performed to detect LINC00847 expression in 55 pairs of LSCC tissues and adjacent tissues. C. The Kaplan-Meier database was used to analyze the relationship between LINC00847 expression and LSCC patients’ survival. *** *P* < 0.001.

Through the analysis of data from GEPIA database (http://gepia.cancer-pku.cn/), it was revealed that LINC00847 expression in LSCC tissue samples was significantly up-regulated (Figure 1(a)). Subsequently, LINC00847 expression in LSCC tissues and cells was examined through qRT-PCR, and it was unveiled that LINC00847 expression in LSCC tissues was markedly up-regulated compared with para-cancerous tissues (Figure 1(b)). As shown in Table 1, high LINC00847 expression was associated with poor differentiation and lymph node metastasis. Additionally, LSCC patients with high LINC00847 expression had a lower survival rate (Figure 1(c)).

**Table 1.** Correlation between clinicopathological features and LINC00847 expression in LSCC

Pathological parameter	Number (n = 55)	LINC00847 expression high (n=27) low (n=28)		χ^2^	*p-*value
Gender				1.114	0.291
Male	37	20	17		
Female	18	7	11		
Age (years)				1.039	0.308
< 60	35	19	16		
≥ 60	20	8	12		
Drinking				0.475	0.490
Yes	30	16	14		
No	25	11	14		
Smoking				3.561	0.0591
Yes	36	21	15		
No	19	6	13		
T staging				2.182	0.139
T1-2	25	15	10		
T3-4	30	12	18		
Differentiation				13.459	0.000*
Low, medium	23	18	5		
High	32	9	23		
Lymph node metastasis				6.557	0.010*
Yes	25	17	8		
No	30	10	20		

*P < 0.05.

### 3.2 Effects of LINC00847 on LSCC cell proliferation, migration, invasion, and cell cycle progression

**Figure 2.**
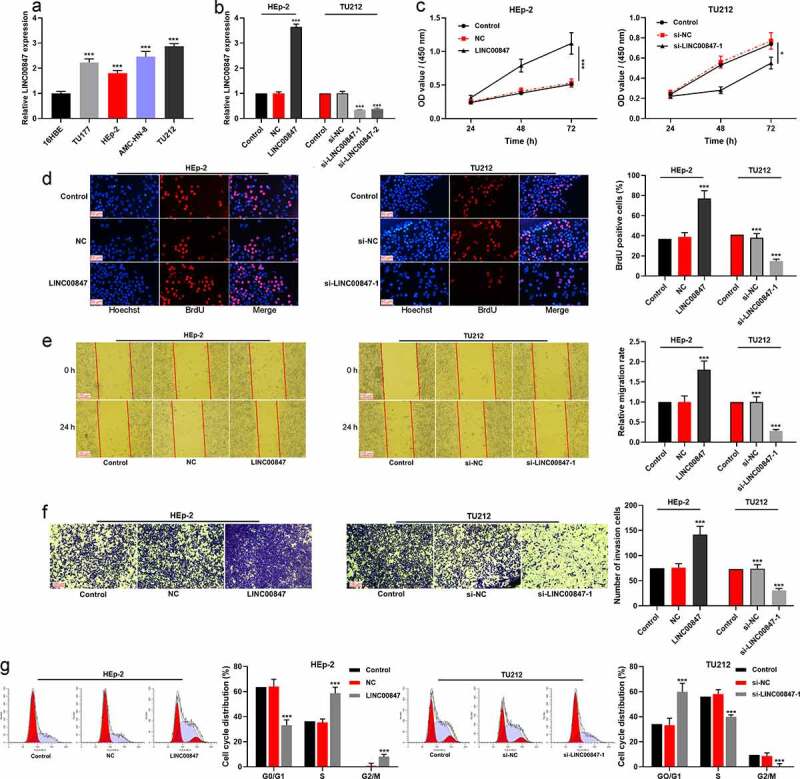
**Regulatory effects of LINC00847 on LSCC cell proliferation, migration, invasion, and cell cycle progression** A. qRT-PCR was conducted to detect LINC00847 expression in human bronchial epithelial cells (16HBE) and LSCC cell lines (TU177, HEp-2, AMC-HN-8, and TU212). B. qRT-PCR was utilized to detect the transfection efficiency of LINC00847 overexpression plasmid, si-LINC00847-1 and si-LINC00847-2. C&D. CCK-8 method and BrdU assay were adopted to detect the effects of LINC00847 overexpression and knockdown on LSCC cell proliferation. E&F. Scratch wound healing assay and Transwell assay were performed to detect the effects of LINC00847 overexpression and knockdown on LSCC cell migration and invasion. G. Flow cytometry assay was conducted for detecting the impacts that LINC00847 knockdown and overexpression on LSCC cell cycle progression. * *P* < 0.05, ** *P* < 0.01, and *** *P* < 0.001.

qRT-PCR showed that LINC00847 expression was remarkably enhanced in LSCC cell lines (HEp-2, AMC-HN-8, TU177, and TU212) compared with 16HBE (Figure 2(a)). In addition, the distribution of LINC00847 in TU212 cells was detected using RNA-FISH assay, and the results indicated that LINC00847 was mainly located in the cytoplasm of TU212 cells (Supplementary Figure 1A). Then, HEp-2 cells were transfected with LINC00847 overexpression plasmids, and si-LINC00847-1 and si-LINC00847-2 were transfected into TU212 cells, and qRT-PCR was used to verify the success of transfection (Figure 2(b)). CCK-8 assay showed that compared with the control group, LINC00847 overexpression significantly promoted HEp-2 cell proliferation while knocking down LINC00847 inhibited TU212 cell proliferation (Figure 2(c)). The results of BrdU assay showed that as opposed to the control group, in the LINC00847 overexpression group, the rate of BrdU-positive cells was markedly increased, whereas in the si-LINC00847-1 group, that rate was noticeably decreased (Figure 2(d)). Next, Transwell and scratch wound healing assays were conducted for examining the effects of LINC00847 on cell migration and invasion, and it was discovered that LINC00847 overexpression dramatically facilitated cell migration and invasion, while knocking down LINC00847 suppressed cell migration and invasion (Figure 2(e-f)). Flow cytometry analysis showed that LINC00847 overexpression facilitated the cell cycle progression from G0/G1 phase to S phase, whereas knocking down LINC00847 could block the cell cycle progression in G0/G1 phase (Figure 2(g)).

### 3.3 LINC00847 directly targets miR-181a-5p

**Figure 3.**
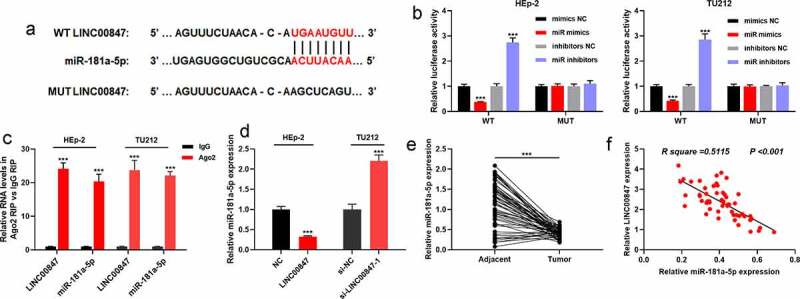
**LINC00847 directly targets miR-181a-5p** A. StarBase database was employed to predict the binding sites between LINC00847 and miR-181a-5p. B. The dual-luciferase reporter assay was carried out to verify the binding sites between LINC00847 and miR-181a-5p. C. RIP assay was utilized to analyze the interaction between LINC00847 and miR-181a-5p. D. qRT-PCR was used to detect the effects of LINC00847 overexpression and knockdown on miR-181a-5p expression. E. qRT-PCR was used to detect miR-181a-5p expression in 55 pairs of LSCC and adjacent tissues. Pearson correlation analysis of the correlation between miR-181a-5p expression and LINC00847 expression in LSCC tissues. *** *P* < 0.001.

To clarify the downstream targets of LINC00847, using the StarBase database, it was revealed that miR-181a-5p was a potential functional target miRNA of LINC00847 (Figure 3(a)). To further verify the targeting relationship between miR-181a-5p and LINC00847, dual-luciferase reporter assay was conducted. It was discovered that transfection of miR-181a-5p mimics suppressed the luciferase activity of WT LINC00847, and transfection of miR-181a-5p inhibitors promoted the luciferase activity of WT LINC00847, while transfection of miR-181a-5p inhibitors or miR-181a-5p mimics did not significantly change the luciferase activity of MUT LINC00847 (Figure 3(b)). Furthermore, RIP assay validated that in comparison to control IgG, LINC00847 and miR-181a-5p were noticeably enriched in Ago2-containing microribonucleoproteins (Figure 3(c)). RNA pull-down assay also showed that miR-181a-5p was remarkably enriched by the Bio-LINC00847-probe rather than Bio-NC-probe or Bio-LINC00847-MUT-probe (Supplementary Figure 1B). Subsequently, qRT-PCR was utilized for examining miR-181a-5p expression in tissues and cells, and it was revealed that LINC00847 overexpression remarkably reduced miR-181a-5p expression, while LINC00847 knockdown significantly elevated miR-181a-5p expression (Figure 3(d)). In comparison with the adjacent tissues, miR-181a-5p expression was down-modulated in LSCC tissues (Figure 3(e)), and Pearson’s correlation analysis showed a negative correlation between miR-181a-5p and LINC00847 expression levels in LSCC tissues (Figure 3(f)).

### 3.4 Impacts of LINC00847 and miR-181a-5p on LSCC cell proliferation, migration, invasion, and cell cycle

**Figure 4.**
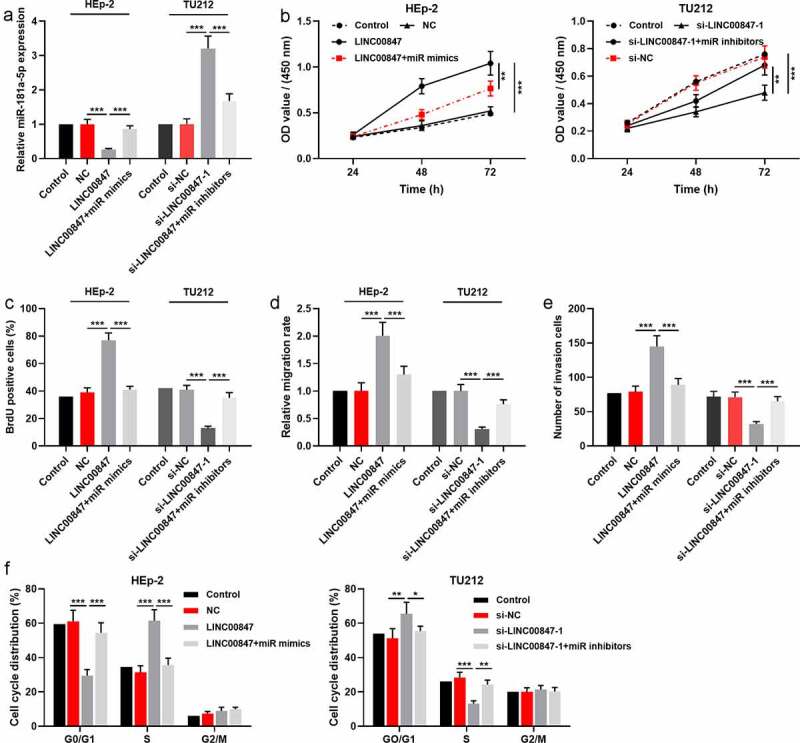
**LINC00847 promotes LSCC cell proliferation, migration, invasion, and cell cycle by adsorbing miR-181a-5p** A. qRT-PCR was employed to detect the transfection efficiency of the co-transfection of LINC00847 overexpression plasmids and miR-181a-5p mimics, and of the co-transfection of si-LINC00847-1 and miR-181a-5p inhibitors. B&C. CCK-8 and BrdU assays were conducted to detect LSCC cell proliferation. D&E. Scratch wound healing assay and Transwell assay were used to detect LSCC cell migration and invasion. F. Flow cytometry assay was conducted for detecting LSCC cell cycle progression. * *P* < 0.05, ** *P* < 0.01, and *** *P* < 0.001.

To further investigate the relationship between miR-181a-5p and LINC00847, HEp-2 cells were co-transfected with miR-181a-5p mimics and LINC00847 overexpression plasmids, and si-LINC00847-1 and miR-181a-5p inhibitors were co-transfected into TU212 cells, which were confirmed by qRT-PCR to be successful (Figure 4(a)). Then, through CCK-8, BrdU, scratch wound healing, Transwell and flow cytometry assays, it was found that as opposed to the control group, LINC00847 overexpression markedly facilitated LSCC cells’ proliferation, migration and invasion, and cell cycle progression, while transfection of miR-181a-5p mimics reversed the above-mentioned effects; knocking down LINC00847 significantly suppressed LSCC cells’ proliferation, migration, and invasion, and blocked the cell cycle, whereas transfection of miR-181a-5p inhibitors reversed these effects (Figure 4(b-f)).

### 3.5 LINC00847 up-modulated ZEB2 expression via adsorbing miR-181a-5p

**Figure 5.**
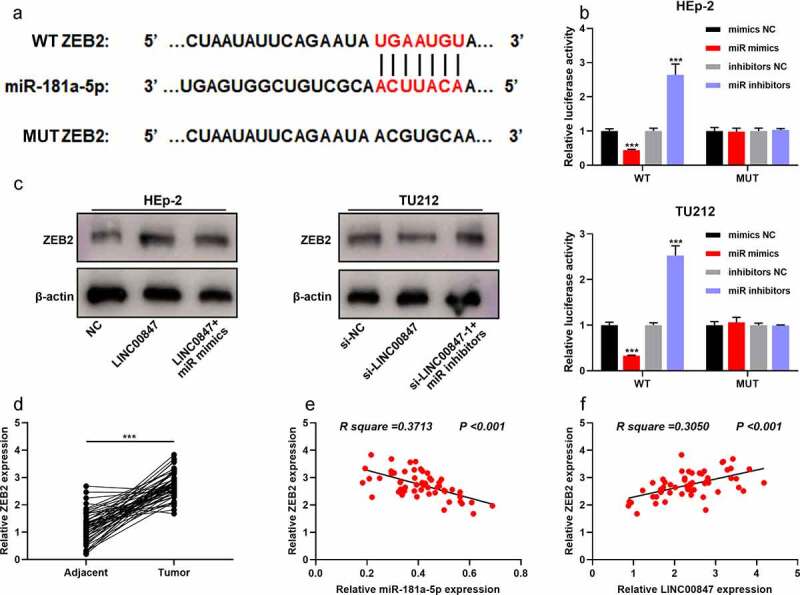
**LINC00847 up-regulates ZEB2 expression by adsorbing miR-181a-5p** A. The online database TargetScan was adopted to predict the binding site between miR-181a-5p and ZEB2 mRNA 3ʹUTR. B. Dual-luciferase reporter assay was performed to verify the binding site between miR-181a-5p and ZEB2. C. Western blot was performed to detect the regulatory effects of LINC00847 and miR-181a-5p mimics on ZEB2 expression. D. ZEB2 mRNA expression was detected by qRT-PCR in 55 pairs of LSCC and adjacent tissues. E&F. Pearson correlation analysis of the correlation among LINC00847, miR-181a-5p and ZEB2 mRNA expressions in LSCC tissues. *** *P* < 0.001.

To further clarify the downstream regulatory mechanism of miR-181a-5p, TargetScan database (http://www.targetscan.org/vert_72/) was searched for predicting the downstream targets of miR-181a-5p, and it was showed that ZEB2 was a downstream target of miR-181a-5p (Figure 5(a)). Dual-luciferase reporter gene assay indicated that miR-181a-5p overexpression inhibited the luciferase activity of WT ZEB2, and miR-181a-5p inhibition promoted the luciferase activity of WT ZEB2, whereas neither miR-181a-5p mimic nor inhibitor significantly affected the luciferase activity of MUT ZEB2 (Figure 5(b)). Western blot suggested that LINC00847 overexpression promoted ZEB2 expression, whereas miR-181a-5p overexpression had the opposite effect (Figure 5(c)); knocking down LINC00847 inhibited ZEB2 expression, whereas miR-181a-5p inhibition had the opposite effect (Figure 5(c)). Besides, miR-181a-5p overexpression inhibited ZEB2 mRNA and protein expression, whereas ZEB2 overexpression counteracted this effect (Supplementary Figure 1C&D). CCK-8 assay showed that the inhibitory effect of miR-181a-5p overexpression on cell viability was reversed by ZEB2 overexpression (Supplementary Figure 1E). The results of the *in vivo* experiment showed that LINC00847 knockdown suppressed TU212 cell xenograft tumor growth in nude mice, while overexpression of ZEB2 counteracted this effect (Supplementary Figure 1F). Through qRT-PCR, it was revealed that compared with the adjacent tissues, ZEB2 mRNA was significantly highly expressed in LSCC tissues (Figure 5(d)), and miR-181a-5p expression and ZEB2 mRNA expression were negatively correlated, while LINC00847 expression and ZEB2 mRNA expression were positively correlated (Figure 5(e-f)).

## 4. Discussion

In the past few years, numerous lncRNAs have been found in mammalian genomes, and the number may exceed the number of protein-coding genes [30,31]. LncRNAs have similar properties to messenger RNAs, can be spliced, and have a 5’ methylcytosine cap and a 3’ poly tail [32,33], but they are not translated into proteins [34]. LncRNAs are located in the nucleus or cytoplasm and regulate gene expression level at transcriptional level and post-transcriptional level [35–37]. Some studies have reported that lncRNAs are strongly associated with the pathogenesis and progression of LSCC. For instance, lncRNA MEG3 expression is decreased in LSCC, and its low expression is linked to advanced clinical stage; MEG3 can increase apoptotic protease activating factor-1 (APAF-1) expression by adsorbing miR-23a to suppress LSCC cell proliferation and induce the apoptosis [38]. LncRNA RGMB-AS1 can facilitate LSCC cell proliferation and invasion by regulating the miR-22/NLRP3 molecular axis [39]. Noteworthily, LINC00847 expression is enhanced in renal cancer cells, and down-regulating LINC00847 expression *in vitro* can block renal cancer cell cycle and induce cell apoptosis [13]. In this study, it was revealed for the first time that LINC00847 expression in LSCC tissues was observably increased as against para-carcinoma tissues, and the high expression was significantly correlated with unfavorable clinical features including lymph node metastasis and poor differentiation. These results suggest that LINC00847 is expected to be an indicator for evaluating the prognosis of LSCC patients. In addition, LINC00847 overexpression could significantly promote LSCC cell proliferation, migration, invasion, and accelerate cell cycle progression while knocking down LINC00847 inhibited the aforementioned biological behaviors of LSCC cells. These data imply that LINC00847 can promote LSCC progression.

MiRNA is also non-coding RNA, which modulates target protein expression through binding to target mRNA 3ʹUTR and facilitating mRNA degradation or inhibiting protein translation [40]. Many studies have shown that abnormal expression of miRNA is associated with cancer (including LSCC) progression [41]. For example, miR-424-5p can promote LSCC cell proliferation, migration, invasion and adhesion by regulating CADM1 expression [42]; miR-632 can regulate GSK3β expression to promote LSCC cell proliferation and colony formation, to promote cell migration and invasion, and to promote the expressions of cell cycle-related proteins cyclin D1 and c-myc [43]. Additionally, miR-181a-5p has been reported to play a role in regulating diverse cancers. For instance, miR-181a-5p expression is lowered in prostate cancer, and up-regulated expression of miR-181a-5p *in vitro* can suppress cell proliferation and induce cell cycle arrest [44]. Importantly, miR-181a-5p can inhibit GATA6 expression, thereby inhibiting LSCC cell migration and inducing the apoptosis [45]; miR-181a-5p also induces LSCC cell apoptosis and inhibits the proliferation and colony formation by targeting NPM1, thus inhibiting LSCC progression [46]. This study confirmed that miR-181a-5p was a downstream target of LINC00847. In addition, miR-181a-5p could weaken the impacts of LINC00847 on LSCC cell proliferation, migration, invasion, and cell cycle progression.

Known as a member of the zinc finger E-box-binding protein (ZEB) family, ZEB2 can participate in modulating many biological processes, for instance, cell proliferation, cell cycle, apoptosis, angiogenesis, epithelial-mesenchymal transition (EMT) process, chemosensitivity, etc [47–49]. ZEB2 dysregulation is observed in many types of tumors, including gastric cancer, colorectal cancer, pancreatic cancer and LSCC [15,50–52]. In LSCC, high ZEB2 expression is significantly associated with the advancing in tumor stage, the decrease in differentiation and the shortening of overall survival [53]; down-regulating ZEB2 expression can notably suppress LSCC cell viability, migration, invasion and EMT, and induce the apoptosis [15]. In addition, ZEB2 can also be regulated by lncRNA and miRNA. For example, lncRNA-CTS can up-regulate ZEB2 expression by adsorbing miR-505 to facilitate CC cell migration, invasion and EMT [54]; lncRNA UCA1 can regulate the miR-203/ZEB2 axis to facilitate gastric cancer cell migration and invasion [55]. Our study validated that miR-181a-5p targeted ZEB2 and negatively modulated its expression, and that LINC00847 overexpression promoted ZEB2 expression, while knocking down LINC00847 inhibited ZEB2 expression. The aforementioned findings suggest that LINC00847 is able to enhance ZEB2 expression via adsorbing miR-181a-5p to facilitate the malignant progression of LSCC.

## 5. Conclusion

To sum up, for the first time, our study reports that LINC00847 expression is up-regulated in LSCC and is closely associated with the poor clinicopathological indicators of LSCC patients. In terms of mechanism, it is validated that LINC00847 facilitates LSCC development by modulating miR-181a-5p/ZEB2 molecular axis. Our study is helpful to clarify the molecular mechanism of LSCC progression.

## Supplementary Material

Supplemental Material

## Data Availability

The data used to support the findings of this study are available from the corresponding author upon request.
