# Supplementary material for: Long intergenic non-protein coding RNA 847 promotes laryngeal squamous cell carcinoma progression through the microRNA-181a-5p/zinc finger E-box binding homeobox 2 axis
Source: Bioengineered. 2022 Apr 17;13(4):9987–10000. doi: 10.1080/21655979.2022.2062531 (PMC9161931; doi:10.1080/21655979.2022.2062531)
Supplement: Supplemental Material [file KBIE_A_2062531_SM0202.zip › supplementary/Supplementary Figureclean.docx]

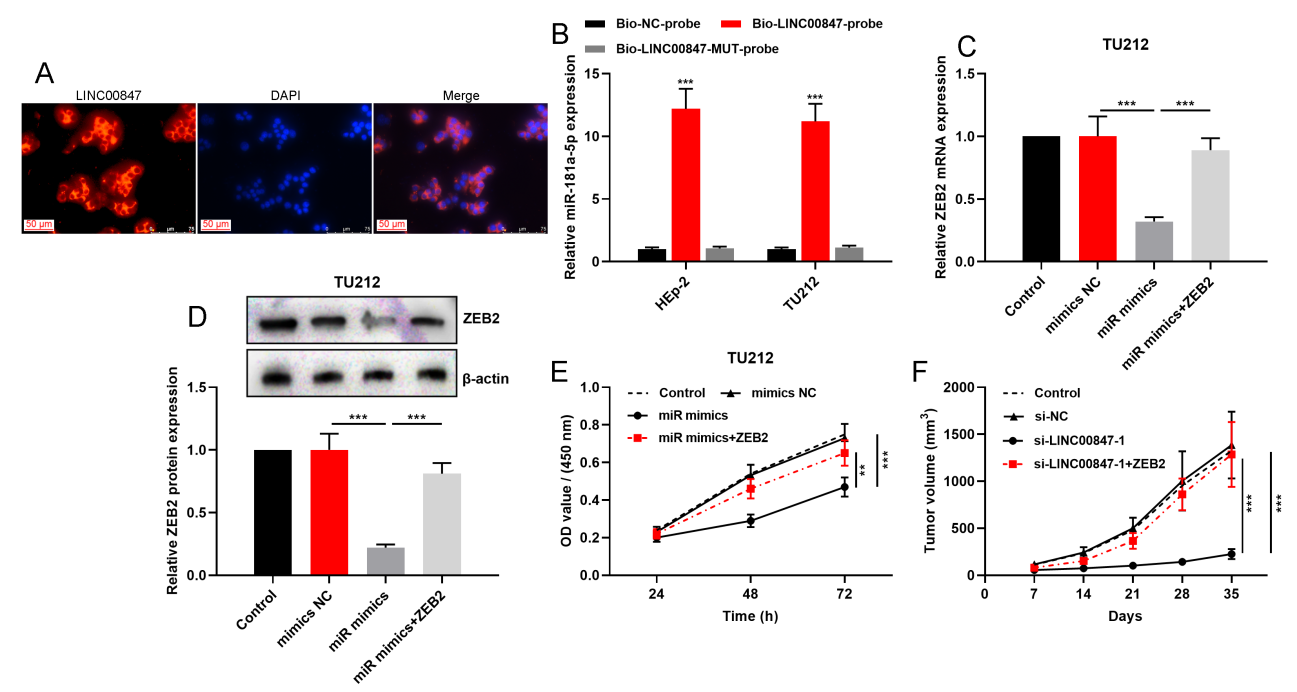


*Supplementary Figure 1*

A. RNA-FISH was performed to determine the distribution of LINC00847 in TU212 cells. Red: LINC00847 was labeled by the RNA probe-LINC00847; blue: cell nuclei were labeled with DAPI. Scale bars = 50 μm.

B. RNA pull down assay was performed to detect the enrichment of miR-181a-5p in Bio-NC-probe, Bio-LINC00847-probe and Bio-LINC00847-MUT-probe group.

C. The transfection efficiency of miR-181a-5p mimics and ZEB2 overexpression plasmid was examined by Western blot and qRT-PCR.

D. The transfection efficiency of miR-181a-5p mimics and ZEB2 overexpression plasmid was examined by Western blot.

E. The regulatory effects of miR-181a-5p and ZEB2 on the viability of TU212 cells were detected by CCK-8 assay.

F. Xenograft model was established using TU212 cells to detect the effects of LINC00847 and ZEB2 on the tumor growth of LSCC *in vivo.*
