# Supplementary figures and images for: Long intergenic non-protein coding RNA 847 promotes laryngeal squamous cell carcinoma progression through the microRNA-181a-5p/zinc finger E-box binding homeobox 2 axis
Source: Bioengineered. 2022 Apr 17;13(4):9987–10000. doi: 10.1080/21655979.2022.2062531 (PMC9161931; doi:10.1080/21655979.2022.2062531)

Figure 5C

Figure 5C


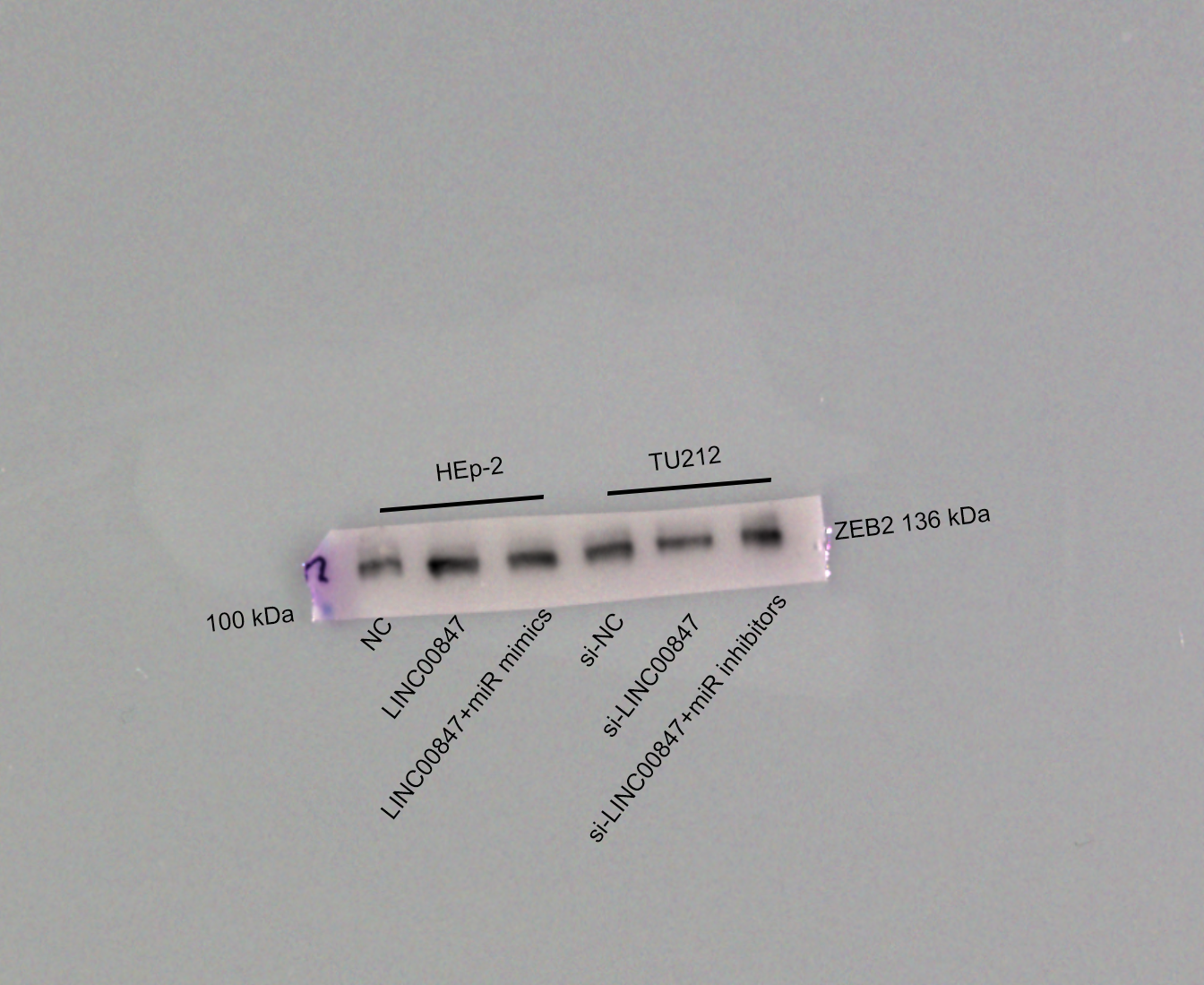

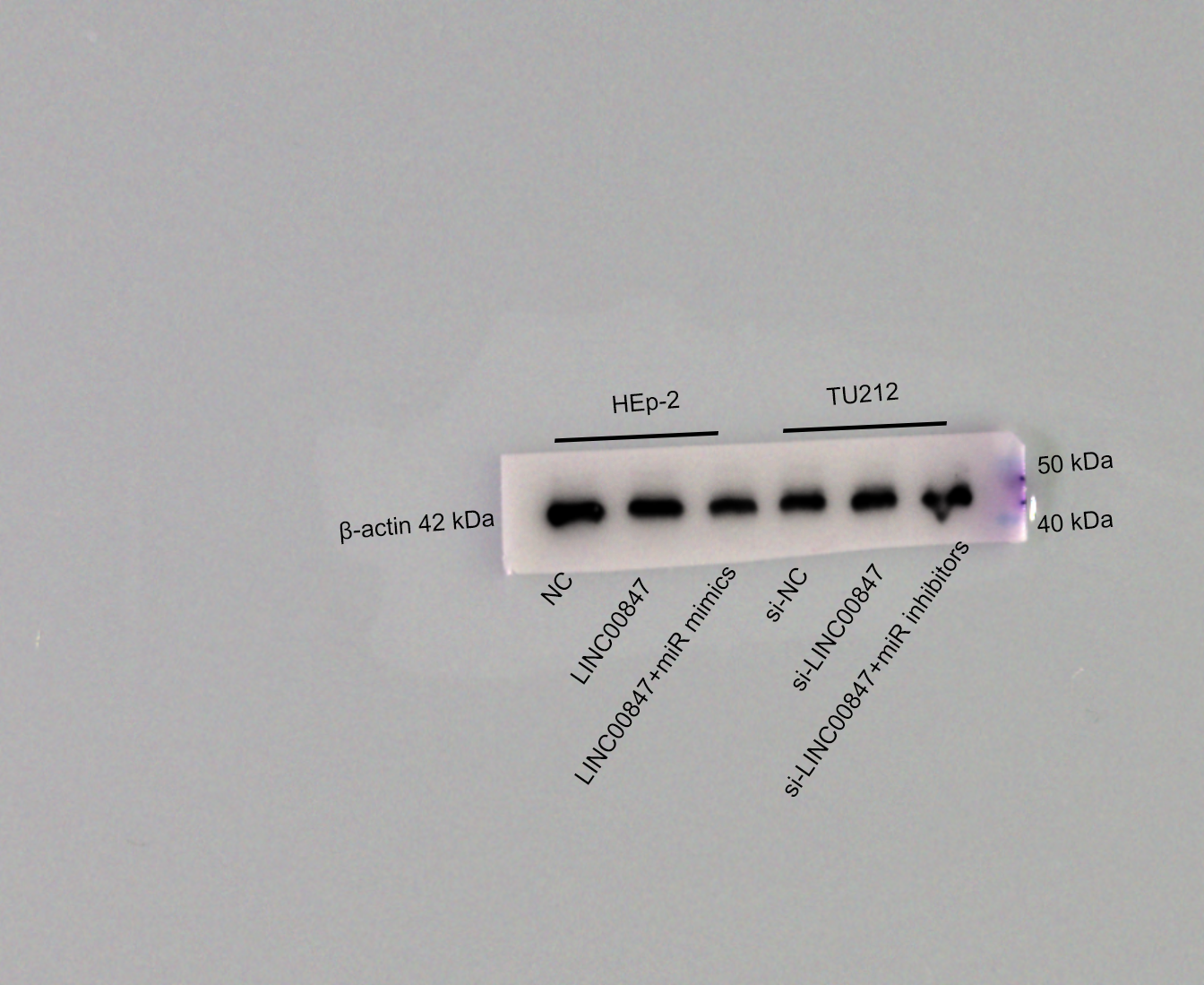


Sup 1D

Sup 1D


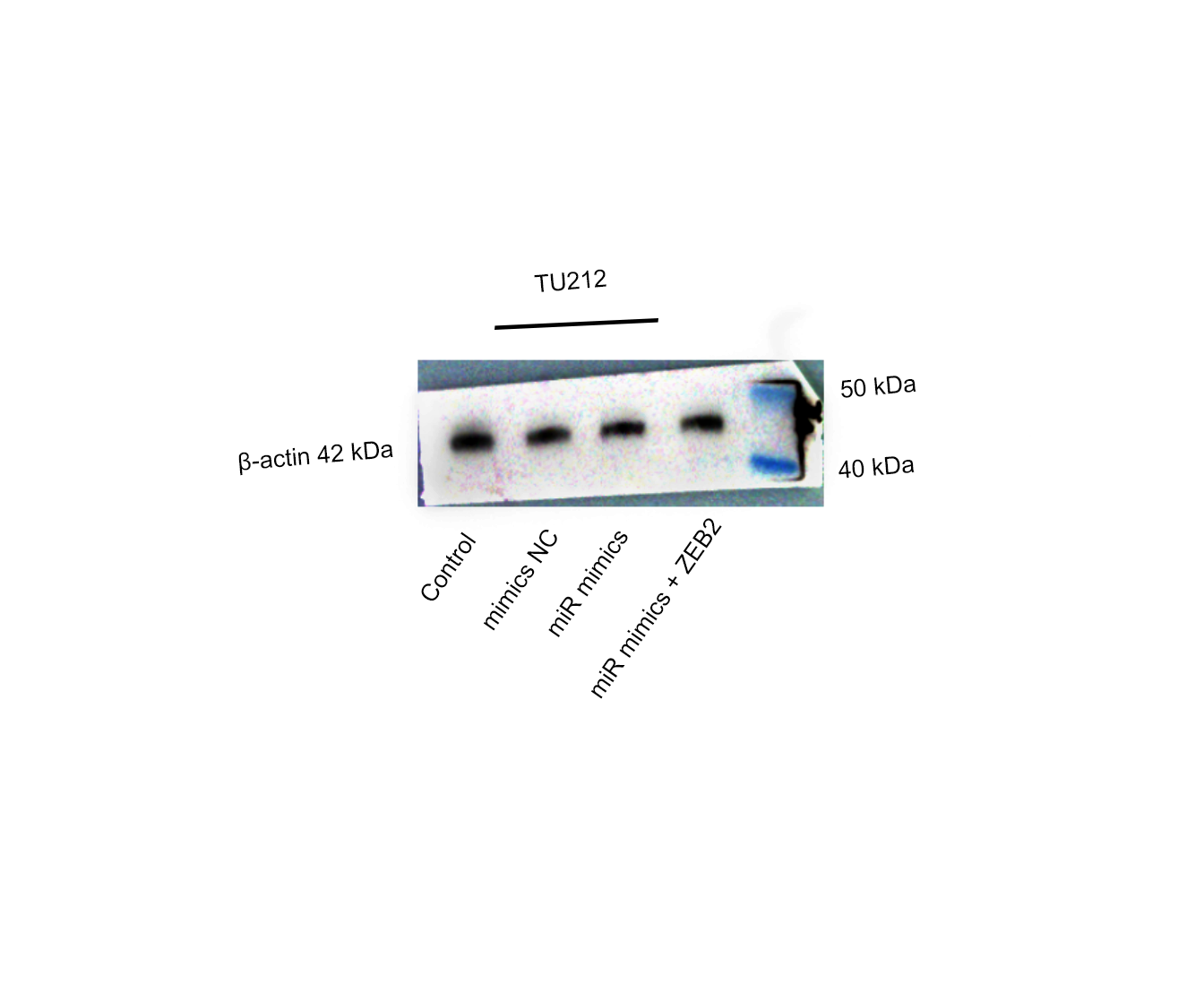

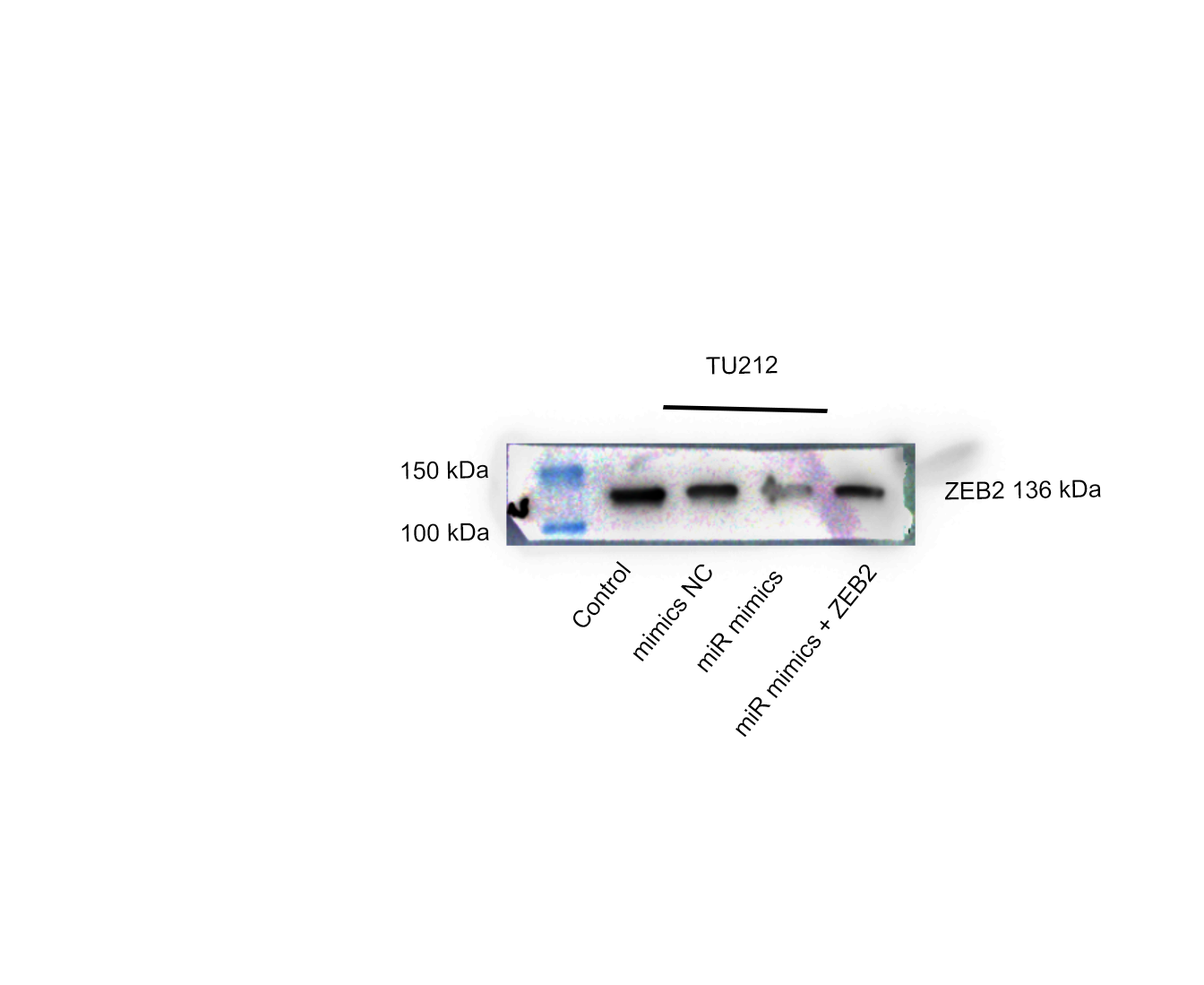

Supplement: Supplemental Material [file KBIE_A_2062531_SM0202.zip › supplementary/Original Western blot results.docx]
